# Association Between Pre-operative BUN and Post-operative 30-Day Mortality in Patients Undergoing Craniotomy for Tumors: Data From the ACS NSQIP Database

**DOI:** 10.3389/fneur.2022.926320

**Published:** 2022-07-19

**Authors:** Yufei Liu, Haofei Hu, Zongyang Li, Yong Han, Fanfan Chen, Mali Zhang, Weiping Li, Guodong Huang, Liwei Zhang

**Affiliations:** ^1^Department of Neurosurgery, Beijing Tiantan Hospital, Capital Medical University, Beijing, China; ^2^Department of Neurosurgery, The First Affiliated Hospital of Shenzhen University, Shenzhen Second People's Hospital, Shenzhen, China; ^3^Shenzhen University Health Science Center, Shenzhen, China; ^4^Department of Nephrology, The First Affiliated Hospital of Shenzhen University, Shenzhen Second People's Hospital, Shenzhen, China; ^5^Department of Emergency, The First Affiliated Hospital of Shenzhen University, Shenzhen Second People's Hospital, Shenzhen, China

**Keywords:** blood urea nitrogen, brain tumor, craniotomy, prognosis, mortality risk

## Abstract

**Objective:**

There is limited evidence to clarify the specific relationship between pre-operative blood urea nitrogen (BUN) and post-operative 30-day mortality in patients undergoing craniotomy for tumors. Therefore, we aimed to investigate this relationship in detail.

**Methods:**

Electronic medical records of 18,642 patients undergoing craniotomy for tumors in the ACS NSQIP from 2012 to 2015 were subjected to secondary retrospective analysis. The principal exposure was pre-operative BUN. Outcome measures were post-operative 30-day mortality. We used binary logistic regression modeling to evaluate the association between them and conducted a generalized additive model and smooth curve fitting (penalized spline method) to explore the potential relationship and its explicit curve shape. We also conducted sensitivity analyses to ensure the robustness of the results and performed subgroup analyses.

**Results:**

A total of 16,876 patients were included in this analysis. Of these, 47.48% of patients were men. The post-operative 30-day mortality of the included cases was 2.49% (420/16,876), and the mean BUN was 16.874 ± 6.648 mg/dl. After adjusting covariates, the results showed that pre-operative BUN was positively associated with post-operative 30-day mortality (OR = 1.020, 95% CI: 1.004, 1.036). There was also a non-linear relationship between BUN and post-operative 30-day mortality, and the inflection point of the BUN was 9.804. For patients with BUN < 9.804 mg/dl, a 1 unit decrease in BUN was related to a 16.8% increase in the risk of post-operative 30-day mortality (OR = 0.832, 95% CI: 0.737, 0.941); for patients with BUN > 9.804 mg/dl, a 1 unit increase in BUN was related to a 2.8% increase in the risk of post-operative 30-day mortality (OR = 1.028, 95% CI: 1.011, 1.045). The sensitivity analysis proved that the results were robust. The subgroup analysis revealed that all listed subgroups did not affect the relationship between pre-operative BUN and post-operative 30-day mortality (*P* > 0.05).

**Conclusion:**

Our study demonstrated that pre-operative BUN (mg/dl) has specific linear and non-linear relationships with post-operative 30-day mortality in patients over 18 years of age who underwent craniotomy for tumors. Proper pre-operative management of BUN and maintenance of BUN near the inflection point (9.804 mg/dl) could reduce the risk of post-operative 30-day mortality in these cases.

## Background

Craniotomies are the cornerstone of intracranial tumor treatment. However, craniotomies with the removal of brain tumors display significant complications and mortality (1, 2). The post-operative 30-day mortality is widely regarded as a predictor of surgical risk after craniotomy (3, 4). The reported 30-day mortality after intracranial tumor surgery is 2.3–3.2% (2, 5, 6). Blood urea nitrogen (BUN), a product of the metabolism of nitrogen compounds, was described to be associated with mortality in various human diseases. Elevated BUN regarding renal dysfunction is associated with the increased risk of incident diabetes (7) and mortality in cardiovascular diseases (8–10). A non-linear correlation between BUN and post-operative 30-day mortality was reported in patients with sepsis (11). BUN has a predictive value of prognostic risk in laparotomy for strangulated small bowel obstruction (12) and post-operative stroke risk after cardiac surgical procedures (13).

Low BUN may indicate insufficient protein intake (14). Inadequate protein intake could result in poor nutritional status. Nutritional status is closely related to the prognosis of cancer patients (15, 16). To date, the relationship between pre-operative BUN and post-operative 30-day mortality has yet to be explored in patients undergoing craniotomies for brain tumors. Thus, this study was designed to examine the relationship between pre-operative BUN and post-operative 30-day mortality in cross-sectional study data from a large US population with brain tumors (age > 18 years). This study may provide guidance for clinical practice by clarifying the quantitative relationship between them.

## Participants and Methods

### Study Design

This cross-sectional study utilized data from the American College of Surgeons National Surgical Quality Improvement Program (ACS NSQIP) database, from records between 2012 and 2015. Our independent variable was pre-operative BUN. The dependent variable was post-operative 30-day mortality.

### Data Source

The data studied were obtained from the ACS NSQIP database originally uploaded by Jingwen Zhang et al. [data from (4)]. The original study was an open access article distributed under a Creative Commons Attribution License, which permits unrestricted use, distribution, and reproduction in any medium, provided the original author and source are credited. Therefore, these data could be used for secondary analysis without infringement on the authors' rights.

### Participants

**Figure 1:**
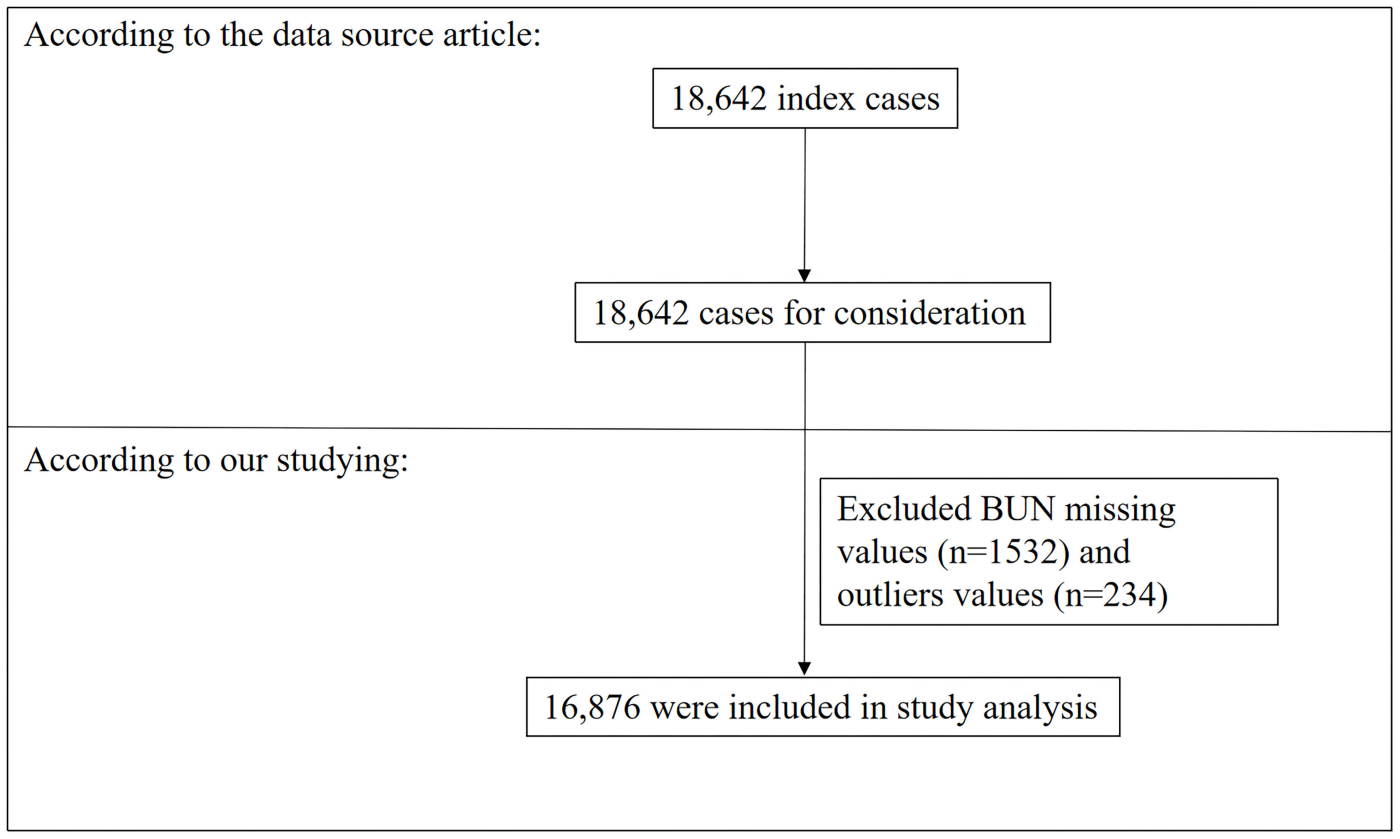
Flowchart of study participant selection.

A total of 18,642 adults with brain tumors were included in the original study. After excluding patients with missing values for BUN (*N* = 1,532) and outliers (defined as values more than ± 3 standard deviations from the mean) (*N* = 234), 16,876 cases were included in our analysis (as shown in Figure 1). Consent forms from participants were not required because our study was based on a secondary analysis of previously collected data and the original personal information was anonymous.

### Variables

#### BUN

The pre-operative BUNs (mg/dl) were recorded as continuous variables in the original study (4). Data collected include pre-operative risk factors (i.e., BUN), comorbidities, procedures performed by the Current Procedural Terminology (CPT) code, and post-operative complications occurring within 30 days of the index operation.

### Post-operative 30-Day Mortality

The post-operative 30-day mortality was defined as mortality that occurred in the first 30 post-operative days (4). NSQIP tracked mortality, readmissions, and reoperation after discharge for the first 30 post-operative days.

### Covariates

In our study, covariates were selected according to our clinical experience and the previous literature. Thus, the following variables were treated as covariates: (1) continuous variables: pre-operative blood test indicators (serum sodium (Na), creatinine (Cr), white blood cell (WBC) count, hematocrit (HCT), platelet (PLT) count), BMI, and duration of the operation and (2) categorical variables: sex (female or male), race (Asian, white, African American, or unknown), age ranges (18–40, 41–60, 60–80, >80 years), smoking status, ventilator dependence, steroid use, pre-operation transfusions, and emergency case status, and a history of diabetes [no, yes (non-insulin-dependent), or yes (insulin-dependent)], severe chronic obstructive pulmonary disease (COPD), renal failure, congestive heart failure (CHF), hypertension, disseminated cancer, American Society of Anesthesiologists physical status classification (ASACLAS), and open wound infection [BMI = weight in kilogram divided by height in meter squared (kg/m^2^)]. More specific details are presented in the original study (4).

### Statistical Analysis

The number of participants with missing values for height, weight, and Na was 594 (3.52%), 299 (1.77%), and 197 (1.16%), respectively. The missing values were replaced by the mean value for statistical analysis.

We stratified the participants by quartiles of BUN. The mean ± SD (normally distributed variables) or median (interquartile range; non-normally distributed variables) was reported for continuous variables, and frequencies and percentages were presented for categorical variables. We used χ^2^-tests (categorical variables), one-way ANOVAs (normally distributed variables), or Kruskal–Wallis *H*-tests (non-normally distributed data) to test for significant differences among different BUN groups. To examine the link between BUN and post-operative 30-day mortality, three distinct univariate and multivariate binary logistic regression models were constructed according to the STROBE statement guidelines, including a non-adjusted model (no covariates were adjusted), a minimally adjusted model (adjusted for sex, race, and age range), and a fully adjusted model (adjusted for sex, race, age ranges, BMI, diabetes, smoke, severe COPD, hypertension, renal failure, dialysis, disseminated cancer, open wound infection, emergency case, bleeding disorders, Na, Cr, HCT, WBC, PLT, ASACLAS, and steroid use; covariates are presented in Table 1). Effect sizes (OR) and their 95% confidence intervals (CIs) were recorded. We adjusted the effect sizes when co-variances were added to the model and the matched hazard ratio was changed by 10% or more (17).

**Table 1:** Baseline characteristics of participants.

**BUN (quartile)**	**Q1 (1.000–12.000)**	**Q2 (12.000–16.000)**	**Q3 (16.000–20.000)**	**Q4 (20.000–42.297)**	***P*-value**
*N* (cases)	3,599	4,598	3,844	4,835	
Sex, *N* (%)					<0.001
Male	1,234 (34.287%)	2,103 (45.737%)	1,996 (51.925%)	2,680 (55.429%)	
Female	2,365 (65.713%)	2,495 (54.263%)	1,848 (48.075%)	2,155 (44.571%)	
Age ranges *N* (%)					<0.001
18−40	1,015 (28.202%)	938 (20.400%)	497 (12.929%)	268 (5.543%)	
41−60	1,703 (47.319%)	2,115 (45.998%)	1,628 (42.352%)	1,601 (33.113%)	
61−80	845 (23.479%)	1,466 (31.883%)	1,610 (41.883%)	2,660 (55.016%)	
>81	36 (1.000%)	79 (1.718%)	109 (2.836%)	306 (6.329%)	
Race, *N* (%)					<0.001
White	2,373 (65.935%)	3,371 (73.314%)	2,867 (74.584%)	3,684 (76.194%)	
Asian	108 (3.001%)	166 (3.610%)	107 (2.784%)	116 (2.399%)	
African American	356 (9.892%)	316 (6.873%)	248 (6.452%)	273 (5.646%)	
Unknown race	762 (21.173%)	745 (16.203%)	622 (16.181%)	762 (15.760%)	
BMI (Mean ± SD)	28.112 ± 6.269	28.387 ± 5.962	28.394 ± 5.985	28.637 ± 5.931	0.001
Serum sodium (Mean ± SD)	138.741 ± 3.079	139.025 ± 3.050	138.829 ± 3.127	138.036 ± 3.413	<0.001
Creatinine (Mean ± SD)	0.738 ± 0.287	0.801 ± 0.229	0.844 ± 0.253	0.956 ± 0.411	<0.001
White blood cells (Mean ± SD)	8.306 ± 3.571	8.636 ± 3.761	9.586 ± 4.337	11.206 ± 4.760	<0.001
Hematocrit (Mean ± SD)	39.629 ± 4.698	40.486 ± 4.610	40.760 ± 4.695	40.248 ± 5.071	<0.001
Platelet (Mean ± SD)	250.178 ± 69.085	242.883 ± 66.475	240.502 ± 67.495	235.682 ± 74.116	<0.001
Diabetes, *N* (%)					<0.001
No	3,331 (92.553%)	4,174 (90.779%)	3,396 (88.345%)	3,988 (82.482%)	
Yes (Non-insulin-dependent)	186 (5.168%)	284 (6.177%)	296 (7.700%)	501 (10.362%)	
Yes (Insulin-dependent)	82 (2.278%)	140 (3.045%)	152 (3.954%)	346 (7.156%)	
Smoking status, *N* (%)					<0.001
No	2,719 (75.549%)	3,709 (80.666%)	3,115 (81.035%)	4,007 (82.875%)	
Yes	880 (24.451%)	889 (19.334%)	729 (18.965%)	828 (17.125%)	
Severe COPD, *N* (%)					<0.001
No	3,468 (96.360%)	4,437 (96.498%)	3,681 (95.760%)	4,510 (93.278%)	
Yes	131 (3.640%)	161 (3.502%)	163 (4.240%)	325 (6.722%)	
Hypertension, *N* (%)					<0.001
No	2,663 (73.993%)	3,130 (68.073%)	2,382 (61.967%)	2,222 (45.957%)	
Yes	936 (26.007%)	1,468 (31.927%)	1,462 (38.033%)	2,613 (54.043%)	
Renal failure, *N* (%)					0.009
No	3,599 (100.000%)	4,598 (100.000%)	3,843 (99.974%)	4,829 (99.876%)	
Yes	0 (0.000%)	0 (0.000%)	1 (0.026%)	6 (0.124%)	
Dialysis, *N* (%)					<0.001
No	3,597 (99.944%)	4,595 (99.935%)	3,840 (99.896%)	4,812 (99.524%)	
Yes	2 (0.056%)	3 (0.065%)	4 (0.104%)	23 (0.476%)	
Disseminated cancer, *N* (%)					<0.001
No	2,980 (82.801%)	3,820 (83.080%)	3,028 (78.772%)	3,334 (68.956%)	
Yes	619 (17.199%)	778 (16.920%)	816 (21.228%)	1,501 (31.044%)	
Open wound infection, *N* (%)					0.048
No	3,566 (99.083%)	4,564 (99.261%)	3,822 (99.428%)	4,782 (98.904%)	
Yes	33 (0.917%)	34 (0.739%)	22 (0.572%)	53 (1.096%)	
Steroid use, *N* (%)					<0.001
No	3,243 (90.108%)	4,066 (88.430%)	3,266 (84.964%)	3,756 (77.684%)	
Yes	356 (9.892%)	532 (11.570%)	578 (15.036%)	1,079 (22.316%)	
Bleeding disorders, *N* (%)					<0.001
No	3,545 (98.500%)	4,518 (98.260%)	3,771 (98.101%)	4,699 (97.187%)	
Yes	54 (1.500%)	80 (1.740%)	73 (1.899%)	136 (2.813%)	
Emergency case, *N* (%)					<0.001
No	3,304 (91.803%)	4,311 (93.758%)	3,602 (93.704%)	4,554 (94.188%)	
Yes	295 (8.197%)	287 (6.242%)	242 (6.296%)	281 (5.812%)	
ASACLAS, *N* (%)					<0.001
No Disturb	67 (1.862%)	68 (1.479%)	43 (1.119%)	33 (0.683%)	
Mild Disturb	1,091 (30.314%)	1,337 (29.078%)	965 (25.104%)	808 (16.711%)	
Severe Disturb	1,942 (53.959%)	2,658 (57.808%)	2,329 (60.588%)	3,146 (65.067%)	
Life Threat	442 (12.281%)	496 (10.787%)	468 (12.175%)	793 (16.401%)	
Moribund	57 (1.584%)	39 (0.848%)	39 (1.015%)	55 (1.138%)	

The non-linear relationship between BUN and post-operative 30-day mortality was addressed using a generalized additive model (GAM) and smooth curve fitting (penalized spline method). If non-linearity was detected, we first calculated the inflection point using a recursive algorithm and then constructed a piecewise binary logistic regression model with one piece on each side of the inflection point. The log-likelihood ratio test was employed to determine the most suitable model for describing the association between BUN and post-operative 30-day mortality.

Several sensitivity analyses were conducted to test the robustness of our results. We converted BUN into a categorical variable according to its quartiles and calculated the *P*-value for each trend to verify the results of using BUN as a continuous variable and examine the possibility of non-linearity. We also explored the potential for unknown confounds in the relationship between BUN and post-operative 30-day mortality by calculating *E*-values (18). As brain tumor patients with dialysis and renal failure may influence the relationship between pre-operative BUN and post-operative 30-day death in this study, we excluded patients with dialysis and (or) renal failure and performed another multivariate analysis to verify the robustness of our findings. All results were reported according to the STROBE statement guidelines (17, 19).

Modeling was performed with EmpowerStats (http://www.empowerstats.com, X and Y Solutions, Inc., Boston, MA) and the statistical software package R (http://www.R-project.org, The R Foundation). *P*-values < 0.05 (two-sided) were considered statistically significant.

## Results

### Baseline Characteristics of Participants

Baseline characteristics of 16,876 participants based on the quartiles of BUN are shown in Table 1. Notably, 47.38% of patients who participated was men. The age distribution proportions were 16.11% (18–40 years), 41.76% (41–60 years), 39.00% (61–80 years), and 3.14% (>81 years). The mean BUN was 16.874 ± 6.648 mg/dl. The post-operative 30-day mortality of the included cases was 2.49% (420/16,876). We assigned participants into subgroups using BUN quartiles as follows: Q1 (1.000–12.000 mg/dl), Q2 (12.000–16.000 mg/dl), Q3 (16.000–19.900 mg/dl), and Q4 (20.000–42.297 mg/dl). Comparing participants with a lower BUN (1.000–12.000 mg/dl), the higher BUN (20.000–42.297 mg/dl) was significantly positively correlated with sex, age range, race, BMI, pre-operative blood test indicators (serum Na, BUN, WBC, HCT, and PLT), diabetes, smoking status, severe COPD, hypertension, renal failure, dialysis, disseminated cancer, open wound infection, steroid use, bleeding disorders, emergency case, and ASACLAS (all *P*-values < 0.05).

### Univariate Analyses Using a Binary Logistic Regression Model of Other Factors

The univariate analysis indicated that patients who were female (OR = 0.772, 95% CI: 0.634–0.941), of unknown race, 41–60 years old, 61–80 years old, >81 years old, BMI, levels of Na, creatinine, WBCs, HCT, and PLTs, and who had diabetes (Insulin-dependent), severe COPD, hypertension, renal failure, disseminated cancer, open wound infection, steroid use, bleeding disorders, emergency cases, were positively associated with post-operative 30-day mortality. In contrast, patients, who were Asian or African American, had creatinine, had diabetes (non-insulin-dependent), were smoking, and had dialysis, were negatively associated with post-operative 30-day mortality (refer to the Supplementary Table for details).

### Multivariate Analyses Using the Binary Logistic Regression Model

To investigate the correlation between BUN and post-operative 30-day mortality, we constructed three models using binary logistic regression models. In the non-adjusted model, an increase of 1 unit of BUN was related to a 6.6% increase in post-operative 30-day mortality (OR = 1.066, 95% CI: 1.053–1.080). The results were statistically significant. In the minimally adjusted model, when the authors only adjusted for sex, race, age ranges, each additional unit of BUN increase could lead to elevated post-operative 30-day mortality by 4.3% (OR = 1.043, 95% CI: 1.029–1.058). The findings on the link between BUN and post-operative 30-day mortality obtained from the model were statistically significant. In the fully adjusted model, each additional BUN unit was accompanied by a 2.0% increase in post-operative 30-day mortality (OR = 1.020, 95% CI: 1.004–1.036). The distribution of CIs indicated that the link between the BUN and the post-operative 30-day mortality obtained by the model was reliable (Table 2).

**Table 2:** The results of the multivariate analysis.

**Exposure**	**Model 1 (OR, 95% CI, *P*)**	**Model 2 (OR, 95% CI, *P*)**	**Model 3 (OR, 95% CI, *P*)**
BUN	1.066 (1.053, 1.080) <0.00001	1.043 (1.029, 1.058) <0.00001	1.020 (1.004, 1.036) 0.01391
BUN (quartile)			
Q1 (1.000–12.000)	Ref	Ref	Ref
Q2 (12.000–16.000)	0.930 (0.654, 1.322) 0.68666	0.827 (0.580, 1.179) 0.29457	0.887 (0.618, 1.273) 0.51657
Q3 (16.000–20.000)	1.381 (0.986, 1.934) 0.06069	1.073 (0.761, 1.513) 0.68573	1.042 (0.731, 1.487) 0.81908
Q4 (20.000–42.297)	2.744 (2.045, 3.684) <0.00001	1.808 (1.326, 2.465) 0.00018	1.363 (0.975, 1.905) 0.07012
*P* for trend	<0.00001	<0.00001	0.01226

*Model 1 (non-adjusted model): not adjusted for any covariates*.

*Model 2 (minimally adjusted model): adjusted sex, race, and age ranges*.

*Model 3 (fully adjusted model): adjusted sex, race, age ranges, BMI, diabetes, smoke, severe COPD, hypertension, renal failure, dialysis, disseminated cancer, open wound infection, emergency case, bleeding disorders, Na, Cr, HCT, WBC, PLT, ASACLAS, and steroid use*.

*OR, odds ratio; 95% CI, 95% confidence interval; Ref, reference*.

### The Non-linearity Addressed by the Generalized Additive Model

**Figure 2:**
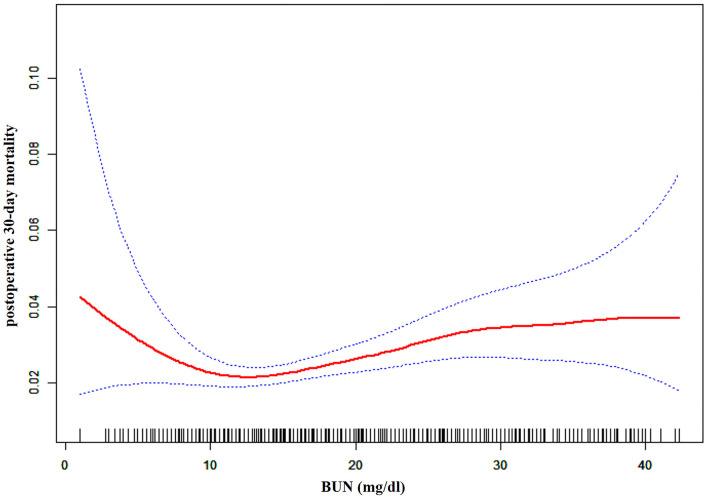
The non-linear relationship between pre-operative BUN and risk of post-operative 30-day mortality.

Through the generalized additive model and smooth curve fitting, we observed that the association between BUN and post-operative 30-day mortality rates was non-linear (Figure 2). Therefore, we fit the data to a piecewise binary logistic regression model that allowed two different slopes. Data were also fitted by a standard binary logistic regression model based on the sensitivity analysis, and the best fit model was selected through the log-likelihood ratio test (Table 3). The *p*-value for the log-likelihood ratio test was <0.05 in our study. Therefore, a piecewise model was used to fit the link between BUN and post-operative 30-day mortality. With a recursive algorithm, we first obtained an inflection point of 9.804 mg/dl and then calculated the effect sizes and CIs to the left and right of the inflection point with the piecewise binary logistic regression model. On the left side of the inflection point, the effect size was 0.832, and the 95% CI was from 0.737 to 0.941. On the right side of the inflection point, the effect size was 1.028, and the 95% CI was from 1.011 to 1.045 (Table 3).

**Table 3:** The results of the piecewise linear regression.

	**Post-operative 30-day mortality** **(OR, 95% CI, *P*-value)**
Infection point of BUN, mg/dl	9.804
<9.804	0.832 (0.737, 0.941) 0.0032
>9.804	1.028 (1.011, 1.045) 0.0009
*P*-value from the log likelihood ratio test	0.003

*This model adjusted for sex, race, age ranges, BMI, diabetes, smoke, severe COPD, hypertension, renal failure, dialysis, disseminated cancer, open wound infection, emergency case, bleeding disorders, Na, Cr, HCT, WBC, PLT, ASACLAS, and steroid use*.

*OR, odds ratio; 95% CI, 95% confidence interval*.

### The Results of Subgroup Analyses

We performed a subgroup analysis to consider other influencing factors that might affect the relationship between pre-operative BUN and post-operative 30-day mortality. We used sex, age ranges, race, diabetes, smoking status, hypertension, disseminated cancer, open wound infection, severe COPD, or white blood cells as the stratification variables to detect the trend of effect sizes in these variables. As summarized in Table 4, there were no significant differences in the relationship in all the different groups (*p*-value for interaction > 0.05).

**Table 4:** Results of interaction analysis and subgroup analysis.

**Characteristic**	**OR (95% CI)**	***P* for interaction**
Sex *N* (%)		0.6765
Male	1.015 (0.994, 1.037)	
Female	1.022 (0.998, 1.046)	
Age ranges		0.4079
18–40	1.007 (0.912, 1.112)	
41–60	1.044 (1.014, 1.076)	
61–80	1.016 (0.995, 1.037)	
>81	1.005 (0.958, 1.055)	
Race		0.3079
White	1.025 (1.006, 1.043)	
Asian	1.014 (0.866, 1.187)	
African American	1.062 (0.991, 1.139)	
Unknown race	0.993 (0.958, 1.030)	
Diabetes		0.9271
No	1.020 (1.003, 1.038)	
Yes (Non-insulin)	1.023 (0.982, 1.065)	
Yes (Insulin)	1.012 (0.970, 1.056)	
Smoking status		0.5169
No	1.017 (1.000, 1.035)	
Yes	1.029 (0.997, 1.062)	
Hypertension		0.4433
No	1.028 (1.003, 1.053)	
Yes	1.016 (0.996, 1.035)	
Disseminated cancer		0.5144
No	1.016 (0.996, 1.037)	
Yes	1.026 (1.003, 1.050)	
Open wound infection		0.3158
No	1.032 (1.015, 1.049)	
Yes	0.970 (0.859, 1.096)	
Severe COPD		0.3998
No	1.028 (1.012, 1.044)	
Yes	1.046 (1.006, 1.088)	
White blood cells *N* (%)		0.8374
WBC <10	1.023 (1.002, 1.044)	
WBC >10	1.020 (0.997, 1.043)	

### Sensitivity Analyses

A series of sensitivity analyses were performed to verify the robustness of our findings.

### The Form of the Categorical Variable of BUN

We converted BUN from a continuous variable to a categorical variable (dividing into groups according to quartiles) and then replaced the previous BUN variable in the model with the categorical-transformed BUN. After BUN was transformed into a categorical variable, the trend of the effect sizes in different groups was equidistant, and the *p*-value for the trend was consistent with the result when BUN was a continuous variable (Table 2).

### *E*-Value

We computed an E-value to assess the sensitivity to unmeasured confounders. The *E*-value was 1.16, which was greater than the relative risk of unmeasured confounders influencing the relationship between pre-operative BUN and post-operative 30-day mortality, suggesting that the unmeasured or unknown confounders had less effect on the relationship between them.

### Sensitivity Analyses After Excluding Brain Tumor Patients With Dialysis and Renal Failure

We excluded brain tumor patients with dialysis and renal failure in other sensitivity analyses. After excluding the participants with dialysis and renal failure, a total of 16,815 cases were included in another multivariate analysis to verify the robustness of our findings. These results suggested that after adjusting the fully confounding factors, pre-operative BUN was also positively associated with post-operative 30-day mortality (OR = 1.027, 95% CI: 1.012–1.043; Table 5). The results obtained from all of the sensitivity analyses indicated the well-robustness of our findings.

**Table 5:** The results of the multivariate analysis after excluding brain tumor patients with dialysis and (or) renal failure.

**Exposure**	**Model 1 (OR, 95% CI, *P*)**	**Model 2 (OR, 95% CI, *P*)**	**Model 3 (OR, 95% CI, *P*)**
BUN	1.068 (1.054, 1.082) <0.00001	1.045 (1.030, 1.060) <0.00001	1.027 (1.012, 1.043) 0.00048
BUN (quartile)			
Q1 (1.000–11.765)	Ref	Ref	Ref
Q2 (12.000–15.966)	0.947 (0.665, 1.348) 0.76189	0.844 (0.591, 1.206) 0.35213	0.879 (0.601, 1.285) 0.50420
Q3 (16.000–19.900)	1.389 (0.989, 1.951) 0.05764	1.085 (0.768, 1.534) 0.64232	1.095 (0.757, 1.582) 0.63013
Q4 (20.000–41.000)	2.780 (2.066, 3.741) <0.00001	1.848 (1.352, 2.525) 0.00012	1.498 (1.067, 2.104) 0.01968
*P* for trend	<0.00001	<0.00001	0.00104

*Model 1 was sensitivity analysis in participants without dialysis and (or) renal failure (non-adjusted model)*.

*Model 2 was sensitivity analysis in participants without dialysis and (or) renal failure. We adjusted sex, race, and age ranges*.

*Model 3 was sensitivity analysis in participants without dialysis and (or) renal failure. We adjusted sex, race, age ranges, BMI, diabetes, smoke, severe COPD, hypertension, disseminated cancer, open wound infection, emergency case, bleeding disorders, ASACLAS, steroid use, Na, Cr, WBC, HCT, and PLT*.

*OR, odds ratio; 95% CI, 95% confidence interval; Ref, reference*.

## Discussion

We explored the association of pre-operative BUN with post-operative 30-day mortality in patients undergoing craniotomies for tumors in ~400 hospitals and academics across the United States in our study. Our results indicated that there was a non-linear relationship between pre-operative BUN and the risk of post-operative 30-day mortality. A threshold effect curve was found as well, and different correlations of BUN on post-operative 30-day mortality were detected on both sides of the inflection point. Curve fitting and threshold effect analysis revealed that for patients with BUN < 9.804 mg/dl, a 1 unit decrease in BUN was related to a 16.8% increase in the risk of post-operative 30-day mortality (OR = 0.832, 95% CI: 0.737, 0.941); for patients with BUN > 9.804 mg/dl, a 1 unit increase in BUN was related to a 2.8% increase in the risk of post-operative 30-day mortality (OR = 1.028, 95% CI: 1.011, 1.045). With 9.804 mg/dl mg/dL as an inflection point for BUN, patients who underwent craniotomies have a remarkably different risk of death.

Previous studies have reported that BUN is considered to be an important factor in predicting patient mortality (11, 20–27). Higher BUN was associated with poor prognosis in critically ill patients admitted to ICU (21, 24), critically ill patients with cardiogenic shock (23), patients with acute myocardial infarction (27), unstable coronary syndromes (25), and primary pulmonary hypertension (26). The prognostic value of BUN to serum albumin ratio was reported in patients with *Escherichia coli* bacteremia (28), ICU patients with lung cancer (29), critically ill patients with acute pulmonary embolism (30), and patients with aspiration pneumonia (31). These studies confirmed the value of BUN in predicting the prognosis of those patients. We share their views that, in general, higher BUN is associated with a poorer prognosis based on our findings. For patients with BUN > 9.804 mg/dl, a 1 unit increase in BUN was related to a 2.8% increase in the risk of post-operative 30-day mortality in our study. Such results may be due to the fact that elevated BUN is generally considered an important indicator of poor prognosis in heart and kidney patients (32–35). Massari et al. demonstrated that BUN was a stronger biomarker of peripheral congestion than the estimated glomerular filtration rate (36). The BUN level can reflect not only the degree of renal impairment but also protein catabolism in the human body (11). Low BUN may indicate insufficient protein intake (14). Inadequate protein intake could result in poor nutritional status. Nutritional status is closely related to the prognosis of cancer patients (15, 16). These reasons may explain our findings that for brain tumor patients with BUN < 9.804 mg/dl, a 1 unit decrease in BUN was related to a 16.8% increase in the risk of post-operative 30-day mortality. Therefore, BUN could be used as a pre-operative quantitative factor to evaluate the prognosis of patients undergoing craniotomy for brain tumors. The proper pre-operative management of BUN could reduce post-operative 30-day mortality in these cases.

Our study has some strengths as follows. (1) It is the large sample size that allows such analysis. Most covariates have complete information, with few missing. (2) To the best of our knowledge, this is the first time to observe the association between BUN with post-operative 30-day mortality in patients undergoing craniotomies for tumors. (3) We found that there was a non-linear relationship between pre-operative BUN and risk of post-operative 30-day mortality; therefore, our analysis has greater clinical value, which previous studies have not explored. (4) We performed interaction analysis and subgroup analysis. (5) We conducted several sensitivity analyses to test the robustness of our results.

Our research has the following shortcomings and needs attention. (1) This was a retrospective study on the national database. (2) As this study was a secondary analysis of published data, we cannot exclude some unmeasured and/or residual confounding factors (e.g., socioeconomic factors, pharmacological treatments, and characteristics of benign and malignant tumors) that could influence the estimated relationship. However, we calculated the *E*-value to quantify the potential impact of unmeasured confounders and found that they were unlikely to explain the results. (3) We could not explore the relationship between pre-operative BUN and long-term outcomes. Despite these limitations, our study was based on data from a large and heterogeneous group of patients in a large catchment area. Thus, the relationships and conclusions postulated in this study remain highly plausible.

## Conclusion

In patients over 18 years of age who underwent craniotomy for intracranial tumors, pre-operative BUN (mg/dl) has specific linear and non-linear relationships with post-operative 30-day mortality. Our study may provide a reference for policy-makers to develop guidelines as to safer levels of BUN for patients preparing to undergo craniotomy for intracranial tumors. Proper pre-operative management of BUN and maintenance of BUN near the inflection point could reduce post-operative 30-day mortality in these cases.

## Data Availability Statement

The original contributions presented in the study are included in the article/Supplementary Material, further inquiries can be directed to the corresponding authors.

## Ethics Statement

The studies involving human participants were reviewed and approved by Clinical Research Ethics Committee of Shenzhen Second People's Hospital. Written informed consent for participation was not required for this study in accordance with the national legislation and the institutional requirements.

## Author Contributions

YL and HH: formal analysis and methodology. YL: investigation. WL, GH, and LZ: supervision. YL, HH, and YH: writing—original draft. ZL, FC, and MZ: writing—review and editing. All authors contributed to the article and approved the submitted version.

## Conflict of Interest

The authors declare that the research was conducted in the absence of any commercial or financial relationships that could be construed as a potential conflict of interest.

## Publisher's Note

All claims expressed in this article are solely those of the authors and do not necessarily represent those of their affiliated organizations, or those of the publisher, the editors and the reviewers. Any product that may be evaluated in this article, or claim that may be made by its manufacturer, is not guaranteed or endorsed by the publisher.

## Abbreviations

BUN: blood urea nitrogen
OR: odds ratio
CI: Confidence Interval
ACS NSQIP: American College of Surgeons National Surgical Quality Improvement Program
SD: standard deviation
Na: serum sodium
Cr: Creatinine
WBC: white blood cell
HCT: hematocrit
PLT: platelet
COPD: chronic obstructive pulmonary disease
CHF: congestive heart failure
ASACLAS: American Society of Anesthesiologists physical status classification
GAM: generalized additive model
ICU: intensive care unit.
